# Microwave Ablation, Radiofrequency Ablation, Irreversible Electroporation, and Stereotactic Ablative Body Radiotherapy for Intermediate Size (3–5 cm) Unresectable Colorectal Liver Metastases: a Systematic Review and Meta-analysis

**DOI:** 10.1007/s11912-022-01248-6

**Published:** 2022-03-17

**Authors:** Sanne Nieuwenhuizen, Madelon Dijkstra, Robbert S. Puijk, Bart Geboers, Alette H. Ruarus, Evelien A. Schouten, Karin Nielsen, Jan J. J. de Vries, Anna M. E. Bruynzeel, Hester J. Scheffer, M. Petrousjka van den Tol, Cornelis J. A. Haasbeek, Martijn R. Meijerink

**Affiliations:** 1 Department of Radiology and Nuclear Medicine, Amsterdam UMC, Location VUmc, De Boelelaan 1117, 1081 HV Amsterdam, the Netherlands; 2 Department of Surgery, Amsterdam UMC, Location VUmc, Amsterdam, the Netherlands; 3 Department of Radiation Oncology, Amsterdam UMC, Location VUmc, Amsterdam, the Netherlands; 4 Department of Surgery, Medisch Centrum Leewarden, Leeuwarden, the Netherlands

**Keywords:** Thermal ablation, Microwave ablation (MWA), Radiofrequency ablation (RFA), Irreversible electroporation (IRE), Stereotactic ablative body radiotherapy (SABR), Colorectal liver metastases (CRLM)

## Abstract

**Purpose of Review:**

Based on good local control rates and an excellent safety profile, guidelines consider thermal ablation the gold standard to eliminate small unresectable colorectal liver metastases (CRLM). However, efficacy decreases exponentially with increasing tumour size. The preferred treatment for intermediate-size unresectable CRLM remains uncertain. This systematic review and meta-analysis compare safety and efficacy of local ablative treatments for unresectable intermediate-size CRLM (3–5 cm).

**Recent Findings:**

We systematically searched for publications reporting treatment outcomes of unresectable intermediate-size CRLM treated with thermal ablation, irreversible electroporation (IRE) or stereotactic ablative body-radiotherapy (SABR). No comparative studies or randomized trials were found. Literature to assess effectiveness was limited and there was substantial heterogeneity in outcomes and study populations. Per-patient local control ranged 22–90% for all techniques; 22–89% (8 series) for thermal ablation, 44% (1 series) for IRE, and 67–90% (1 series) for SABR depending on radiation dose.

**Summary:**

Focal ablative therapy is safe and can induce long-term disease control, even for intermediate-size CRLM. Although SABR and tumuor-bracketing techniques such as IRE are suggested to be less susceptible to size, evidence to support any claims of superiority of one technique over the other is unsubstantiated by the available evidence. Future prospective comparative studies should address local-tumour-progression-free-survival, local control rate, overall survival, adverse events, and quality-of-life.

## Introduction

Colorectal cancer (CRC) is one of the most common cancers worldwide and the second leading cause of cancer-related mortality, with almost 1.850.000 new cases worldwide and 881.000 deaths in 2018 [1]. Colorectal liver metastases (CRLM) will develop in 25–30% of these patients during the course of their disease and is the main cause of death in CRC patients [2–5]. When left untreated, the 5-year overall survival (OS) rate is dismal, with survival rates around 0–3% [6–8]. Although systemic therapy alone clearly improves survival, the only treatments that can provide long-term disease control or in a subset of patients even cure, are local eradication of the tumour.

Following resection of CRLM, 5-year survival rates of 40–55% can be achieved [3–5, 9–12]. Unfortunately, only 20–30% of patients are considered eligible for partial hepatectomy [3, 4, 13]. Induction chemotherapy can downstage another 10–30% to resectable disease [13–16]. Although generally accepted guidelines are lacking, unresectability of CRLM can be roughly defined as follows: (1) an insufficient volume and function of the future liver remnant after resection, (2) inability to spare the arterial or portal venous blood supply to or the venous or biliary drainage from the future remnant, due to the anatomical location of the lesion(s), (3) an impaired general health status and/or serious cardiopulmonary comorbidities, and (4) an inaccessible abdominal cavity due to extensive previous abdominal surgery.

In the last two decades several radical intent thermal and non-thermal ablative therapies to treat unresectable CRLM emerged. The most well-known are radiofrequency ablation (RFA), microwave ablation (MWA), irreversible electroporation (IRE), and stereotactic ablative body radiotherapy (SABR) [17–24].

There is an ample amount of studies that have shown needle-guided thermal ablation to be effective and safe in the treatment of CRLM ≤ 3 cm [17]. After a median follow-up of 9.7 years, the EORTC-CLOCC trial reported a superior OS of RFA plus chemotherapy over chemotherapy alone (*HR* = 0.58; 95%*CI* 0.38–0.88) with an 8-year OS of 35.9% vs*.* 8.9% [25]. The efficacy of thermal ablation is even being compared to resection in CRLM < 3 cm to prove non-inferiority in the ongoing RCT COLLISION [26]. Conversely, for larger (> 3 cm) CRLM, the primary technique efficacy decreases exponentially, manifesting in higher rates of local tumour progression for all techniques [27–33].

The radiation oncology community has suggested SABR to represent a feasible alternative as local treatment option for a limited number of unresectable CRLM. Although SABR can be effective to establish local control, a trade-off exists between tumour control and collateral damage to surrounding tissue and structures [34–36]. As the efficacy is unaffected by the proximity of large blood vessels and less affected by lesion size and a difficult-to-reach anatomical location, authors have suggested SABR as an alternative to thermal ablation for perivascular, sub-diaphragmatic, and larger CRLM [37, 38].

IRE is a relatively new non-thermal ablative method, where cell death is caused by using high-voltage electric pulses that induce permanent disruption of the membrane [39]. It is thought to be a safe ablation method for tumours adjacent to vascular and biliary structures because it spares the extracellular matrix and as a result preserves critical tubular structures [40].

Extrapolating treatment results of small-sized CRLM, local ablative therapies are also often presumed to prolong survival for unresectable intermediate-size CRLM (3–5 cm). However, given the exponential decrease in local efficacy with increasing lesion size, this presumption requires validation. To ensure patients receive the optimal treatment method, knowledge about the preferred local ablative technique is indispensable. This multidisciplinary systematic review and meta-analysis critically assess and compare the outcomes of local treatment in patients with unresectable intermediate-size CRLM treated with the most widely used thermal and non-thermal ablation techniques.

## Methods

This systematic review and meta-analysis was written according to the Preferred Reporting Items for Systematic Reviews and Meta-Analyses (PRISMA) statement and PICO (patients, interventions, comparisons, outcomes) protocol [41].

### Search

A literature search was performed in the databases PubMed and Embase from January 1st 2008 till November 11th 2020. Keywords used in the search were as follows: colorectal liver metastases, microwave ablation, radiofrequency ablation, stereotactic body radiotherapy, and irreversible electroporation. The full search strategy is presented in appendix 1. The subsequent PICO question was used for the search strategy: P(population): patients with intermediate-size CRLM; intervention: RFA, MWA, IRE, and SABR with or without systemic therapy; comparison: systemic therapy alone; outcome: *critical* endpoints were local-tumour-progression-free survival/local control (LTPFS/LC), complications/toxicity, overall survival (OS), and *important* endpoints were disease-free survival (DFS) and quality of life. The interventional oncology society prefers the use of the term LTPFS (to describe the time from the initial treatment to the first recurrence, regardless of whether the recurrence was reablated), where the radiation oncology society prefers the use of the term local control [42]. Conference abstracts, reviews, meta-analyses, and studies not concerning humans were excluded.

### Study Selection

The abstracts retrieved by this literature search were independently screened by two authors (SN and RP). If the abstracts appeared to adhere to the in- and exclusion criteria, a full-text evaluation was performed. The references of relevant publications were reviewed. References appearing eligible were also submitted to a full-text evaluation. Manuscripts also containing information on efficacy and safety of primary liver carcinoma and non-colorectal liver metastases were allowed if they reported their data on CRLM separately. Studies were excluded if they did not report on at least one of the abovementioned outcome measures distinctly for intermediate size CRLM and if the sample size was less than five. Discrepancies between authors were resolved by consensus.

### Data Extraction

Two authors (SN and MD) extracted the data from the included studies. This concerned the following variables: name author, publication year, years of inclusion, total number of patients, and number of patients with CRLM 3–5 cm, whether patients received prior local treatment of the liver, presence of extrahepatic disease, size of CRLM, amount of CRLM 3–5 cm and/or ≥ 3 cm, treatment modality, and concomitant resections with thermal ablation. The collected data pertaining to study outcomes were for example median follow up, dose and fractions in SABR and biologically equivalent dose (BED10), local control, LTPFS, complications/toxicity, DFS, OS, and quality of life. This data was checked by a third author (RP). In case of discrepancies, these were discussed and resolved by consensus. Additional data of subgroups with intermediate size CRLM was requested and collected from authors that reported results of the comparison of SABR to thermal ablation.

### Data Analysis

Quality assessment criteria per study were based on clinical criteria, such as the included number and specific reporting of intermediate-size CRLM, the population, and the outcome measures used. Pooled analyses were allowed if results from studies were sufficiently similar with regards to these criteria. Studies potentially sufficient to perform meta-analysis were assessed and a random effects model was used to account for statistical heterogeneity. Analysis with the Mantel–Haenszel method was performed to calculate risk ratios (RR) of local tumour progression. Review Manager 5.3 was used to perform the meta-analysis.

### Guidelines

CRLM guidelines were searched using Guideline Central and Guidelines International Network databases.

## Results

**Fig. 1:**
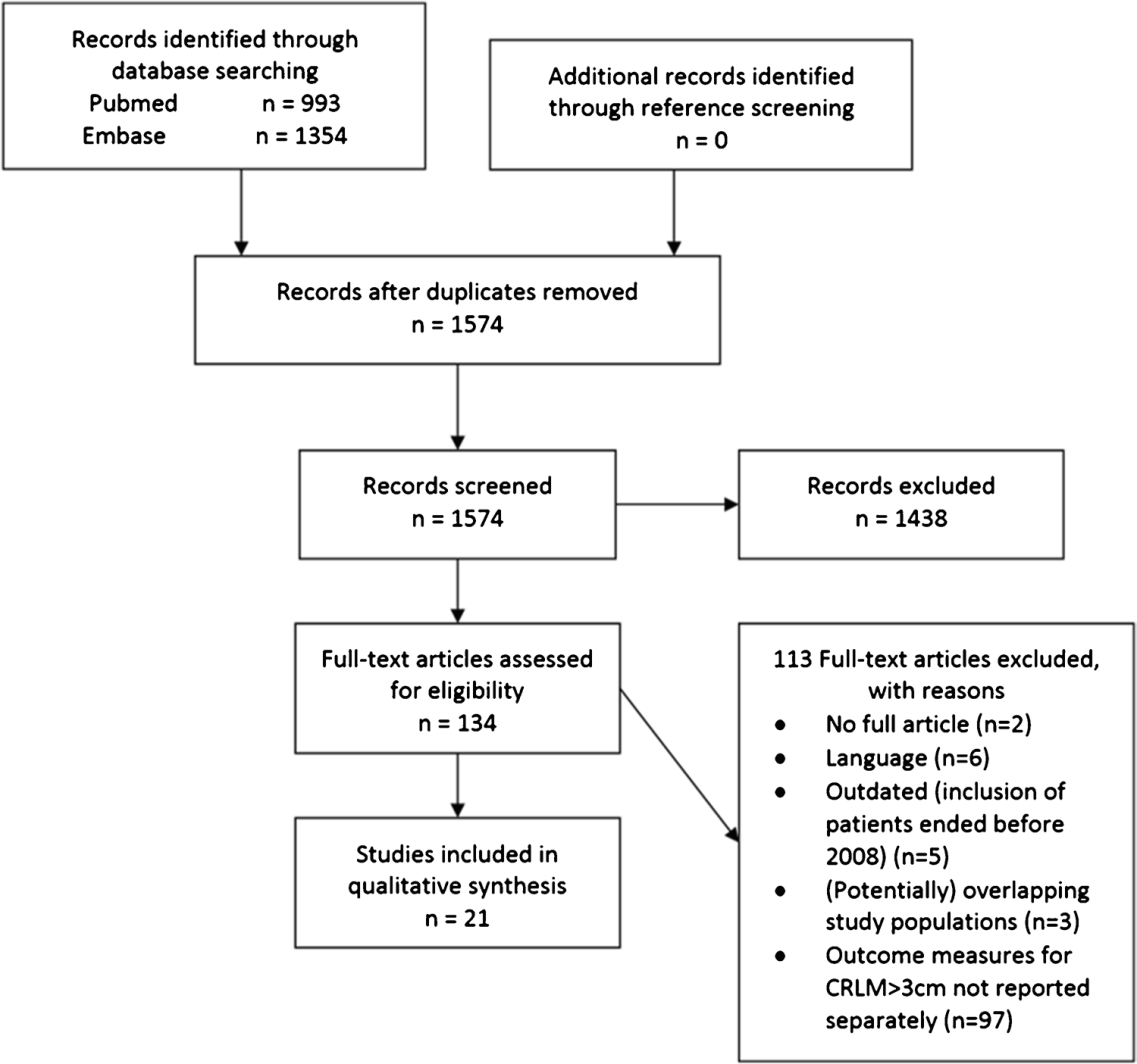
Flowchart of systematic search and selection according to PRISMA

**Fig. 2:**
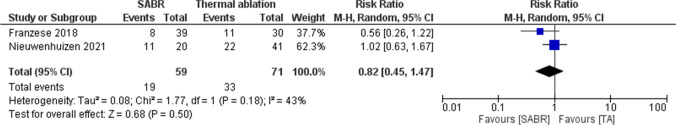
Risk ratio of local tumour progression comparing SABR to thermal ablation (TA)

The search strategy yielded 1685 abstracts after removal of duplicates. After screening the abstracts for eligibility, 151 articles remained for full-text analysis, of which 124 were excluded. This left 27 articles that met our inclusion criteria for qualitative synthesis and 2 articles for quantitative synthesis with meta-analysis (see flowchart in Figs. 1 and 2). Very few publications reported on the outcomes of intermediate-size CRLM (3–5 cm) specifically. Therefore, we allowed publications reporting on the outcomes of CRLM ≥ 3 cm. Series that discontinued including patients before 2008 were excluded, due to the likelihood of outdated results.

### Study Characteristics

There were no randomized controlled trials on ablative treatment methods for intermediate-size CRLM. Of 27 included articles, 20 retrospective series [27, 43–61], and 1 prospective cohort [62] reported on thermal ablation for CRLM > 3 cm: 14 on RFA [27, 43, 45–49, 51, 53, 56–59, 62], 5 on MWA [44, 52, 54, 60, 61], and 2 on both RFA and MWA [50, 55]. One phase II trial [35] and two retrospective series [63, 64] report the outcome of SABR for CRLM > 3 cm. One study reported outcomes for intermediate-size CRLM treated with IRE [65]. Two retrospective series compared SABR to thermal ablation and were included in the meta-analysis [66, 67]. All publications were issued between 2011 and 2020. In the absence of comparative studies, a formal meta-analysis could not be performed. The study population (patient, disease, and lesion characteristics), the use of periprocedural systemic therapy, and oncological outcome measures were highly variable and heterogeneously reported.

## Thermal Ablation

### Patient and Lesion Characteristics

At per patient level, eleven studies reported on 323 patients with at least one ablated CRLM > 3 cm [45–49, 51, 53, 54, 58, 59, 62] (see Table 1). Although simultaneous resections of concomitant resectable CRLM were allowed in 6 studies, none reported outcomes specifically for ablated intermediate-size CRLM with versus without concomitant partial hepatectomy [27, 44, 45, 48, 55, 57]. Half of the studies stated whether patients had received prior focal liver treatment(s) (range 9.1–100%) [27, 43, 46, 50, 51, 53, 56–59]. Extrahepatic disease was allowed in 11 studies [27, 45–47, 51–53, 55–59, 62], disallowed in 5 [43, 48, 49, 54, 60], and not reported in 3 [44, 50, 61]. On a per-lesion basis, 18 studies reported on 760 ablated CRLM > 3 cm: 544 with RFA; 160 with MWA; 56 RFA or MWA [27, 43–50, 52–61].

**Table 1:** Overview of included studies reporting on thermal ablation

Author/year	Type of study	Yrs of inclusion	MWA/ RFA	No pts in tot	No pts CRLM > 3 cm	Age* yrs	Lap/open/perc *	EHD*	Prior local treatment of liver *	Concurrent surgery*	Median FU in months *
Bale/2011 [43]	Retro	2005–2011	RFA	63	-	Med 66	Perc	No	38% of pt	No	25
Eng/2015 [44]	Retro	2009–2013	MWA	33	-	Med 61	Open	-	-	28 pt (85%)	17
Erten/2020 [61]	Retro	2014–2019	MWA	94	-	Mean 61.6	Lap/open	-	-	-	18
Fan/2016 [62]	Prosp	2003–2010	RFA	49	18	-	-	Yes	-	-	-
Gwak/2011 [45]	Retro	2004–2008	RFA	35	10	Med 62	Perc 26pt/open 9 pt	7 pt 20%	No	9 pt (26%)	31
Hamada/2012 [46]	Retro	2002–2010	RFA	84	31	Med 64.6	Perc	23 pt 27%	21 pt (25%)	No	26
Jiang/2019 [47]	Retro	2012–2016	RFA	76	22	-	Perc	40 pt 53%	-	No	32
Kennedy / 2012 [48]	Retro	2000–2010	RFA	130	46	Med 65	Lap	No	-	42 pt (32%)	42
Kim / 2011 [49]	Retro	1996–2008	RFA	177	14	Mean 60.4	Perc/open	No	-	-	41
Liu/2017 [50]	Retro	2004–2013	RFA/ MWA	101	-	Mean 58.2	Perc	-	25 pt, (25%)	No	-
Mao/2019 [51]	Retro	2006–2016	RFA	61	25	Med 59	Perc	8pt 13.1%	61 pt (100%)	No	29
Nielsen/2013 [27]	Retro	2000–2010	RFA	128	-	Mean 62.6	Perc/open	Yes	12 pt (9%)	64	36
Qin/2018 [52]	Retro	2013–2017	MWA	137	-	Mean 54.9	Perc	34 pt 25%	-	No	18
Shady/2015 [53]	Retro	2002–2012	RFA	162	26	-	Perc	51 pt 31%	116 pt (72%)	No	55
Shi/2020 [54]	Retro	2010–2017	MWA	210	68	Mean 59	Perc	No	-	No	48
Takahashi/2018 [55]	Retro	2011–2014	RFA/ MWA	105	-	-	Lap	Yes	-	24 pt (23%)	MWA 17 RFA 18
Valls/2015 [56]	Retro	2005–2012	RFA	59	-	Mean 64.1	Perc	Yes	59 pt (100%)	-	25
Veltri/2012 [57]	Retro	1996–2009	RFA	248	-	Med 67	Perc 243 pt/open 19 pt	51 pt (20%)	102 pt (41%)	19 pt (8%)	19
Wang/2020 [58]	Retro	2013–2018	RFA	85	37	Mean 59	Perc	22 pt (26%)	20 pt (24%)	No	30
Wang/2020 [59]	Retro	2012–2016	RFA	80	26	Mean 59	Perc	28 pt (35%)	12 pt (15%)	No	51
Zhang/2016 [60]	Retro	2009–2014	MWA	199	-	Med 60	Perc	No	-	No	30

*Of total amount of patients

### Overall Survival

#### Colorectal Liver Metastases 3–5 cm

Seven studies reported on OS in patients with at least one intermediate-size CRLM [43, 45, 51, 54, 58, 60, 62]. Median survival ranged 24–39 months [43, 45, 51, 54, 58, 60, 62]. Fan et al. reported the lowest median OS of 24 months [62]. However, in this study patients received cytoreductive RFA with palliative intent in salvage setting. Excluding the outlying results from Fan et al., OS ranged 26–39 months. The 1-, 2-, 3-, and 5-year OS ranged 73–92% [43, 54, 58, 62], 41–72% [43, 54, 58, 62], 20–40% [43, 45, 54, 58, 62], and 10–36% [43, 45, 54, 62], respectively.

#### Colorectal Liver Metastases > 3 cm

Median OS ranged 21.7–37 months in seven retrospective series [27, 43, 46, 48, 53, 57, 59]. The lowest median OS was reported by Veltri et al. [57], a relatively old study that included patients over a longer period of time from 1996 to 2009. More than 40% of their study population had received prior local hepatic treatment and almost 20% of patients presented with extrahepatic disease. The 1-, 2-, 3-, and 5-year OS ranged 74–93%, 30–70%, 20–34%, and 8–31% [48, 49, 53, 57, 59]. See Table 2 for an overview of the survival outcomes.

**Table 2:** Overview of OS outcomes in thermal ablation

Author	Lesion size (range) cm *	No. CRLM 3–5 cm	No. CRLM > 5 cm	No. CRLM > 3 cm	Median OS in months		1 yr OS 3–5 cm	2 yr OS 3–5 cm	3 yr OS	5 yr OS
					3–5 cm	> 3 cm				
Bale [43]	2 (0.5–13)	36	23	59	32	> 3 cm: 31 > 5 cm 29	86%^	72%^	36%^	36%^
Fan [62]	NS (till 5 cm)	-	-	-	24		73%^	41%^	20%^	10%^
Gwak [45]	2.4 (1–5)	-	-	-	Mean 39		-	-	40%	27%
Hamada [46]	2.3 (0.5–9.0)	-	-	35		31	-	-	-	-
Kennedy [48]	2.9 (1–8)	-	-	46	-	29	> 3 cm 93%	> 3 cm 70%^	> 3 cm 34%	> 3 cm 8%
Kim [49]	2.1 (0.5–6.2)	-	-	14	-	-	> 3 cm 84%^	> 3 cm 53%^	> 3 cm 31%^	> 3 cm 31%^
Mao [51]	2.7 (0.9–4)	-	-	-	32	-	-	-	-	-
Nielsen [27]	2.2 (0.2–8.0)	49	20	69	-	37	-	-	-	-
Shady [53]	1.8 (0.5–5.7)	-	-	32		25	> 3 cm 88%^	> 3 cm 50%^	> 3 cm 26%^	> 3 cm 18%^
Shi [54]	2.7 (till 5 cm)	68	-	-	26	-	92% ^	55%^	32%^	20%^
Veltri [57]	2.5 (NS)	-	-	137	-	21.7	> 3 cm 74%^	> 3 cm 39%^	> 3 cm 30%^	> 3 cm 14%^
Wang [58]	2.8 (0.8–5)	52	-	-	26	-	90% ^	42% ^	33%^	-
Wang [59]	2.5 (1–6.4)	-	-	32	-	22	> 3 cm 80%^	> 3 cm 30%^	> 3 cm 20%^	> 3 cm 10%^
Zhang [60]	3 (1–5)	51 (4–5 cm)	-	-	36	-	-	-	-	-

*NS*, not stated

*All-size CRLM included in study

^Percentages retrieved and estimated from OS curves

### Complications and Quality of Life

None of the studies reported the complication rate or the effect of thermal ablation on quality of life specifically for patients with CRLM > 3 cm. Irrespective of lesion size studies reported a major complication rate of 2–17% for percutaneous ablation [43, 46, 47, 50, 52, 53, 56]. Most reported major complications were: pleural effusion, pneumothorax, hepatic abscess, hepatic hematoma, perihepatic bleeding, or ileal perforation. Both Qin et al. and Veltri et al. did not find a correlation between the development of complications and lesion size [52, 57]. Qin et al. found a mean lesion size of 1.8 cm vs 1.5 cm for patients with versus without complications (*p* = 0.101) [52]. Similarly, Veltri et al. found a mean size of 2.7 cm in both groups [57].

### Disease-Free Survival, Local-Tumour-Progression-Free Survival, and Local Control

#### Colorectal Liver Metastases 3–5 cm

Two retrospective series reported DFS [45, 60]. Gwak et al. reported a median DFS of 19 months [45] and Zhang et al. a median DFS of 12 months for patients with CRLM of 4–5 cm [60]. One prospective cohort found a median DFS of 15 months [62]. In four retrospective series, LTP rate varied between 25 and 62% with a median follow up time of 25–36 months [27, 43, 51, 58]. Eventual local control following repeat-ablations was not reported specifically for intermediate-size CRLM. See Table 3 for an overview of the efficacy of thermal ablation.

**Table 3:** Overview of efficacy outcomes of thermal ablation

Author	Lesion size (range) cm *	No. CRLM 3–5 cm	No. CRLM > 3 cm	LTP 3–5 cm	LTP > 3 cm	1 yr LTPFS	2 yr LTPFS	DFS/LTPFS (in months)
Bale [43]	2 (0.5–13)	36	59	11%	-	-	-	DFS > 3 cm 12 DFS > 5 cm 11
Eng [44]	NS (till 5.5)	-	7	-	14%	-	-	-
Erten [61]	NS (0.2–6.6)	-	21	-	19%	-	-	-
Fan [62]	NS (till 5 cm)	-	-	-	-	-	-	Med DFS 3–5 cm: 15
Gwak [45]	2.4 (1–5)	-	-	-	-	-	-	Mean DFS 3–5 cm 19, 3-yr 20% 5-yr 10%
Hamada [46]	2.3 (0.5–9.0)	-	35	-	69%	35%	17%	-
Jiang [47]	2.3 (0.9–5.7)	-	33	-	-	67%	62%	-
Kennedy [48]	2.9 (1–8)	-	46	-	20%	-	-	-
Kim [49]	2.1 (0.5–6.2)	-	14	-	-	-	-	DFS rate 23%
Liu [50]	2.1 (0.7–6.0)	-	23	-	65%	-	-	-
Mao [51]	2.7 (0.9–4)	-	-	25% per tumour, 28% per pt	-	-	-	-
Nielsen [27]	2.2 (0.2–8.0)	49	69	27%	-	-	-	-
Qin [52]	1.5 (0.5–6.7)	12	13	-	38%	-	-	-
Shady [53]	1.8 (0.5–5.7)	-	32	-	78%	36%^	25%^	Med LTPFS 6
Takahashi[55]	≥ 3–NS	-	33	-	45%	69%^	40%^	-
Valls [56]	3–5.8	-	25	-	52%	-	-	-
Wang [58]	2.8 (0.8–5)	52	-	62%	-	60%^	39%^	-
Wang [59]	2.5 (1–6.4)	-	32	-	-	-	-	Med LTPFS 9
Zhang [60]	3 (1–5)	51 (4–5 cm)	-	-	-	-	-	Med DFS 4–5 cm 12

*NS*, not stated

*Of total amount of patients

^Percentages retrieved from graphs

#### Colorectal Liver Metastases > 3 cm

Bale et al. [43] reported a median DFS of 12.4 months from stereotactic RFA. Shady et al. found a median LTPFS of 6 months [53] and Wang of 9 months [59]. Kim et al. found a 5-year DFS rate of 23% [49]. LTP was reported by nine retrospective series and ranged 14–78% with a median follow up time of 17–55 months [44, 46, 48, 50, 52, 53, 55, 56, 61]. The 1- and 2-year LTPFS varied between 34.8–69% and 17.4–62%, respectively [46, 47, 53, 55, 59].

## Stereotactic Ablative Body Radiotherapy

### Patient and Lesion Characteristics

Strict adherence to the inclusion criteria resulted in two retrospective series, as most SABR series do not report separate results based on tumour type and tumour diameter > 3 cm [63, 64]. Doi et al. compared SABR with a conventional fractionated schedule and included 24 patients in total, 15 patients with 21 CRLM > 3 cm and 16 patients (66.7%) with a history of focal hepatic resection(s) and/or thermal ablation(s) [63] (see Table 4). Joo et al. included 70 patients in total, half of the study population had received prior local hepatic treatment, and 19 patients (27%) presented with extrahepatic disease [64]. It was not stated how many patients had intermediate size CRLM.

**Table 4:** Overview of included studies reporting on SABR

Author/ year	Type of study	Yrs of inclusion	Treatment modality	No pts in tot	No pts CRLM > 3 cm	Age* yrs	Dose, fractions, (BED10)	EHD*	Prior local treatment of liver *	Lesion size (range) cm	Median FU in months *
Doi/ 2017 [63]	Retro	2007–2014	LINAC	24	15	64 med	45.0–72.0 Gy, 4–33 fr (71.7–115.5 Gy)	-	16 pt (66.7%)	3.5 (0.7–11.69)	16.5
Joo/2017 [64]	Retro	2007–2014	LINAC	70	-	65 med	30–60 Gy, 3–5 fr (58.4–180 Gy)	19 pt (27%)	35 pt (50%)	2.9	34.2
*Scorsetti/ 2015 *[35]	*Phase II*	*2010*–*2012*	*LINAC*	*42*	*27pt^*	*67 mean*	*45.6*–*85.7 Gy/3fr (262.5 Gy)*	*11 pt (26%)*	*21 pt (50%)*	*3.5 (1.1*–*5.4)*	*24*

*Of total amount of patients

^27 patients with cumulative GTV ≥ 3 cm, not actual lesion size > 3 cm

To collect more data, one prospective phase II trial that studied the efficacy of SABR for 27 CRLM patients with a *cumulative* gross tumour volume (GTV) diameter > 3 cm unsuitable for surgery and thermal ablation was eventually added [35]. Cumulative GTV diameter here means either at least 1 CRLM > 3 cm or multiple smaller CRLM with a cumulative size > 3 cm. Twenty-four CRLM > 3 cm were included. In this study, 11 patients (26%) had extrahepatic disease (EHD) and half of the patients had undergone prior focal liver treatment(s).

### Overall Survival

No study reported OS specifically for CRLM 3–5 cm. Doi et al. reported results both for SABR as for non-ablative radiotherapy and found a median OS of 45 months for patients with at least one CRLM > 3 cm [63]. Conversely, for patients with small-size CRLM ≤ 3 cm, they found a median OS of 27 months [63]. Scorsetti et al. reported a 1-, 2-, and 3-year OS from SABR of 68, 40, and 17%, respectively, for patients with CRLM > 3 cm [35] (Tables 5 and 6).

**Table 5:** Overview of OS and Local Control for SABR

Author	No. CRLM 3–5 cm	No. CRLM > 5 cm	No. CRLM > 3 cm	Median OS > 3 cm	1 yr OS	2 yr OS	3 yr OS	LC > 3 cm	1 yr LC	2 yr LC	LTPFS
Doi [63]	13	8	21	45 mo	-	-	-	-	3–5 cm 50.4% > 5 cm 71.4%	3–5 cm 10.5% > 5 cm 26.8%	15 mo
Joo [64]	-	-	42	-	-	-	-	BED < 132 Gy 67%, BED > 132 Gy 90%	-	-	
*Scorsetti *[35]	*-*	*-*	*24*	*-*	> *3 cm 68%*	> *3 cm 40%*	*17%^*	*-*	*-*	*-*	

^Percentages retrieved from graphs

**Table 6:** Overview of studies comparing SABR to thermal ablation for intermediate size CRLM

Author/ year	Type of study	Yrs of inclusion	No pts SABR/TA	Age * yrs	Median size SABR/TA	Local tumour progression SABR/TA	Median time to local tumour progression SABR/TA	Dose range SBAR	Median FU in months *
Franzese/2018 [66]	Retro	2009–2016	39/30	73	36.5/34.0 cm	20.5%/36.7%	20.0/13.9 months	50.25–75 Gy	24.5
Nieuwenhuizen/2021 [67]	Retro	2005–2011	20/41	63	38.0/44.0 cm	55.0%/53.7%	9.0/6.0 months	40–60 Gy	29.3

*TA*, thermal ablation

*LTPFS*, local tumour progression free survival

*Of total cohort of the study

### Toxicity and Quality of Life

No studies reported the complication rate or the effect of SABR on quality of life for patients with CRLM > 3 cm. Two studies reported no grade ≥ 3 toxicity [35, 64]. Scorsetti et al. found grade 2 acute toxicity in 78% of the study population (55% fatigue, 25% transient hepatic transaminase increase, 12% nausea) [35]. One series reported 2/24 patients with grade 3 toxicity, 1 patient with grade 3 γ-glutamyl transpeptidase (GGT) elevation, and 1 patient with grade 3 GGT and blood bilirubin elevation presumably caused by cholangitis due to a recurrent tumour [63].

### Disease-Free Survival and Local Control

Doi et al. found a 1- and 2-year local control of 50.4% and 10.5% for intermediate-size CRLM and 71.4% and 26.8% for large-size CRLM > 5 cm, respectively [63]. Joo et al. reported a local control for CRLM > 3 cm that correlated with the delivered radiation dose (BED < 132 Gy vs*.* ≥ 132 Gy): 67% vs 90% (*p* = 0.06).

## Irreversible Electroporation

### Patient and Lesion Characteristics

The search resulted in one retrospective series specifically reporting on treatment of intermediate-size CRLM [65]. Fruhling et al. reported on 30 patients in total, of which nine patients had 9 CRLM of 3–4 cm in size. More than half of the patients had received previous local treatment(s) of the liver and all patients were treated by percutaneous IRE. Median follow-up was 22.3 months.

To extend data on IRE for CRLM > 3 cm we included the final results of an as of yet unpublished prospective multicentre phase IIb single-arm study (COLDFIRE-2 trial) where 51 patients were treated with IRE in 62 procedures. Although currently under review, the trial protocol was previously published [68], the results have been presented at ECIO 2019 in Amsterdam, and the outcomes are available as online abstract [69]. Twenty-one (27.6%) out of the 76 IRE-treated CRLM were 3–5 cm in size.

### Overall Survival

Fruhling et al. reported a median OS from IRE for intermediate-size CRLM of 19.7 months [65]. Meijerink et al. reported a median OS from IRE of 32.4 months (95% *CI* 19.2–45.6 months), although they did not report median OS specifically for the subgroup of patients with intermediate-size CRLM.

### Complications and Quality of Life

Fruhling et al. reported four complications in nine patients after IRE of intermediate-size CRLM. Three patients with CTCAE grade I/II complications (episode of shortness of breath, of increased blood pressure and ECG changes during IRE and chest pain requiring morphine) and one patient with a CTCAE grade III complication, namely, a portal vein and biliary duct stricture in the IRE ablated zone. A stent was placed for the portal vein stricture and a percutaneous trans-hepatic cholangiography (PTC) drainage catheter was placed for the biliary duct stricture. Meijerink et al. did not report complications for CRLM 3–5 cm and both series did not report the effect of IRE on quality of life [65].

### Disease-Free Survival, Local-Tumour-Progression-Free Survival, and Local Control

DFS was not reported specifically for CRLM > 3 cm. After a median follow up of 22.3 months, in five out of nine patients (55.6%), local-tumour-progression was detected [65]. Meijerink et al. did not find a significant difference in LTPFS between small- and intermediate-size CRLM (HR 1.72; *CI* 0.73–4.06; *p* = 0.22) [69].With a minimum follow-up of 1 year, median per-patient and per-tumour LTPFS was not reached. Including repeat procedures, local control was eventually realized in 74% (37/50) of patients.

## Comparison of SBAR to Thermal Ablation

### Local Tumour Progression

Franzese et al. performed a propensity score–based comparison of SABR to MWA in 135 patients with CRLM with freedom from local progression (FFLP) as primary endpoint [66]. Stratified analysis by lesion size showed that SABR improved FFLP in patients with lesions > 3 cm and FFLP was similar for both treatment techniques in patients with lesions ≤ 3 cm. Additional data collection showed FFLP specifically for intermediate-size CRLM, suggesting a benefit in local control of SABR compared to MWA in the treatment of larger lesions. After at least 1 year of follow-up, local tumour progression was reported in 8 of 39 CRLM for SABR and 11 of 30 CRLM for MWA of intermediate-size lesions.

Nieuwenhuizen et al. performed a multivariate analysis of thermal ablation compared to SABR for unresectable CRLM to evaluate local tumour progression in the prospective AmCORE registry [67]. Subgroup analyses were performed for larger size lesions (> 3 cm) and additional data collection showed local tumour progression in 11/20 tumours following SABR and 22/41 tumours following thermal ablation with at least 1 year of follow-up.

Overall comparison of local tumour progression following SABR and thermal ablation showed no significant difference (*p* = 0.50).

## Guidelines

Full-text analysis was performed for 12 guidelines [70–81]. One guideline included recommendations for CRLM > 3 cm: the UK National Institute for Health and Care Excellence (NICE) guideline stated that “there is controversy over the indication for RFA, most operators will no longer consider lesions > 4 cm in diameter for treatment” [71]. All other guidelines either did not report on RFA, MWA, SABR, or IRE at all, or they did not state recommendations for CRLM > 3 cm, or they did not state size limitations.

## Discussion

Currently, the preferred treatment method for unresectable intermediate-size CLRM for patients, in whom downstaging or (further) downsizing systemic therapy failed, remains unknown. This systematic review and meta-analysis aimed to collect evidence regarding local ablative therapies to treat unresectable intermediate-size CRLM and to provide a comparison of the most well-known ablative techniques. Literature to reliably assess the oncological outcome was scarce for all treatment options. A substantial shortcoming was the lack of randomized controlled trials comparing treatment methods. In addition, apart from one prospective cohort [62] and one phase II trial [35], virtually, all included studies were retrospective series, with only two of the studies making a comparison between treatment options for intermediate-size CRLM. Furthermore, the reported oncological outcomes, the study population, and the timing of interventions with regard to periprocedural systemic chemotherapy were highly heterogeneous, making it impossible to draw any conclusion.

The majority of publications on thermal ablation concerned RFA. However, for larger-size tumours, recently, preference has started to shift towards newer generation MWA systems or tumour-bracketing multiprobe ablation techniques as potentially superior alternatives to conventional RFA [82, 83]. Presumed benefits of MWA over RFA are consistently higher intratumoural temperatures, faster heating, shorter procedure time, larger ablation volumes, and less susceptibility to the “heat-sink” effect at the cost of a somewhat higher biliary tract complication rate [84–86]. Although few studies compared RFA to MWA for patients with CRLM, several retrospective cohorts reported lower local recurrence rates following MWA compared to RFA, 6% vs. 20% (*p* < 0.01) [19], 10% vs. 20% (*p* = 0.02) [55], 8.6% vs. 20.3% (*p* = 0.07) [87], respectively. In this review, LTP rate at median follow-up after the first ablation ranged 11–78% for RFA [27, 43, 46, 48, 51, 53, 56, 58] and 14–38% for MWA [44, 52, 61]. Although this seems to suggest a preference of MWA for CRLM > 3 cm, the number of MWA treated tumours was low (*n* = 41). A substantial part of the included publications on thermal ablation was relatively old. Consequently, recent advances in technique and improved awareness of the necessity to expand and confirm tumour-free margins following thermal ablation are inadequately represented [53].

For SABR, merely three articles met the inclusion criteria, and all reported different oncological outcome measures. Hence, no conclusions could be drawn regarding efficacy of SABR for intermediate-size CRLM. Many articles describing results for mixed disease and not for CRLM separately could not be included, because metastases deriving from different primary cancers or different organs containing colorectal metastases can have variable responses [88–96]. Several articles were excluded because they presented hazard ratios regarding small versus intermediate-size CRLM but did not report the actual outcomes per size-subgroup, or they reported on the size of CRLM in volumes and not diameter [34, 90, 97].

Two articles met the inclusion criteria for meta-analysis after additional data collection [66, 67]. No difference in local tumour progression was found between SABR and thermal ablation. Two excluded publications compared SABR to thermal ablation for hepatic metastases [98, 99], without specifying outcomes for intermediate-size CRLM. Stintzing et al. compared single session robotic radiosurgery (RRS) to percutaneous RFA in 2 × 30 patients and matched them for size (mean 33–34 mm) and number of lesions [98]. They found that patients treated with RRS had a longer LTPFS compared to patients treated with RFA (34.4 vs. 6.0 months; *p* < 0.001), recurrence rates were similar (67 vs. 63%), and there was a trend towards prolonged median OS for RFA treated patients (34.4 vs 52.3 months; *p* = 0.06). A retrospective cohort by Jackson et al. compared SABR to RFA in 161 patients with liver metastases [99]. SABR demonstrated a superior FFLP compared to RFA, especially for hepatic metastases ≥ 2 cm. There was no difference in median OS (25.9 months for RFA vs. 24.5 months for SABR). These studies, compared to the included studies in meta-analysis, imply a superior local control of SABR compared to thermal ablation for larger-size lesions. However, only comparing local control rates following one ablative procedure seems unjust when comparing a repeatable technique (RFA, MWA) with a technique that usually does not allow for retreatment (SABR). No studies reported a direct comparison of thermal ablation to SABR with regard to periprocedural complications and toxicity for intermediate-size CRLM, though both techniques are associated with an exceptionally low mortality and morbidity rate. Given the comparable overall reported mortality of 0.16% for thermal ablation [100] and 0.5% for SABR [101] (with 3/656 patients mistakenly published as 0.004%) and given the comparable serious adverse event rate of 4–5% for thermal ablation and 9% for SABR [100, 101]. Because both ablative probes and ionizing radiation will potentially result in collateral morbidity by invading surrounding healthy tissue, we prefer to refrain from using the term non-invasive for SABR.

Only two studies concerning IRE were included in this review. This low number can be explained by the relative novelty of this technique and because it is generally a niche indication for CRLM unsuitable for resection and thermal ablation due to close proximity to biliary or vascular structures [40]. Interestingly, the results of the prospective phase II trial (COLDFIRE-2) did not reveal a difference in 1-year LTPFS for small-size versus intermediate-size CRLM, which may indicate that IRE, where electrodes bracket tumours, is less susceptible to differences in size [102].

A recent multidisciplinary consensus document concerning resectability and ablatability criteria for liver only colorectal metastases did not provide strict recommendations for unresectable intermediate-size CRLM due to a lack of evidence and also stated that the exact roles of SBRT and IRE in the treatment of unresectable CRLM need to be further investigated [103].

Although systematically acquired, the results of this systematic review and meta-analysis should be judged with restraint, as only a limited amount of studies could be included, with poor quality and heterogeneous study populations. There is a high risk of publication bias due to the inclusion of mainly retrospective observational studies.

## Conclusion

There are no randomized controlled trials or comparative studies on local treatment for patients with intermediate-size unresectable CRLM. Heterogeneity of the reported oncological outcomes and study populations reduced the amount of obtained data suitable for pooled assessment. Although long-term disease control was described in subsets of patients in all series, there is a lack of studies directly comparing RFA to MWA or to SABR or IRE. No hard conclusions or recommendations can be drawn and further prospective research is necessary to determine what local treatment option, if any, is preferable for intermediate-size unresectable CRLM, preferably in the setting of randomized controlled trials. Therefore, we strongly support the ongoing trials, the COLLISION-XL trial *NCT04081168* (unresectable colorectal liver metastases: stereotactic body radiotherapy versus microwave ablation — a phase II randomized controlled trial for CRLM 3–5 cm), an RCT in Denmark for CRLM < 4 cm *NCT03654131* (stereotactic body radiation therapy vs microwave ablation for colorectal cancer patients with metastatic disease in the liver), and an RCT in Italy for CRLM < 4 cm *NCT02820194* (a trial on SABR versus MWA for inoperable colorectal liver metastases). Hopefully, the results of these trials will clarify and define the role of local ablative methods for the curative intent treatment of permanently unresectable intermediate-size CRLM.

## Data Availability

Not applicable.

## Appendix 1

Pubmed search:

("Colorectal Neoplasms"[Mesh] OR ((colorectal*[tiab] OR colon*[tiab] OR rectal*[tiab] OR rectum[tiab] OR sigmoid) AND (neoplas*[tiab] OR cancer*[tiab] OR carcinoma*[tiab] OR tumour[tiab] OR tumours[tiab] OR tumor[tiab] OR tumors[tiab] OR metasta*[tiab] OR malig*[tiab]))) AND ("Liver Neoplasms"[Mesh] OR ((liver[tiab] OR hepatic*[tiab]) AND (cancer*[tiab] OR neoplas*[tiab] OR tumour[tiab] OR tumours[tiab] OR tumor[tiab] OR tumors[tiab] OR carcinoma*[tiab] OR metasta*[tiab] OR malig*[tiab]))) AND (“Radiosurgery”[Mesh] OR "Microwaves"[Mesh] OR “Electroporation”[Mesh] OR “Radiofrequency ablation”[Mesh] OR stereotactic body radiation therap*[tiab] OR stereotactic body radiotherap*[tiab] OR stereotactic radiotherap*[tiab] OR stereotactic ablative body radiotherap*[tiab] OR stereotactic ablative body radiation therap*[tiab] OR SBRT[tiab] OR SABR[tiab] OR SBR[tiab] OR irreversible electroporation OR IRE OR electroporation OR electropermeabilization OR electrocoagulation OR microwave ablati*[tiab] OR MWA[tiab] OR microwave thermosphere ablati*[tiab] OR RFA[tiab] OR ((radiofreq*[tiab] OR radio-freq*[tiab] OR thermal*[tiab]) AND (ablat*[tiab]))).

Embase search:

('colon cancer'/exp OR 'colon cancer' OR 'rectum cancer'/exp OR 'rectum cancer' OR (((colorectal OR colon* OR rect* OR sigmoid) NEAR/5 (neoplas* OR cancer* OR carcin* OR tumour* OR tumor* OR metasta* OR malig*)):ab,ti,kw)) AND ('liver metastasis'/exp OR (((liver OR hepatic*) NEAR/5 (cancer* OR tumour* OR tumor* OR neoplas* OR malign* OR carcinom* OR metastas*)):ab,ti,kw)) AND ('radiosurgery'/exp OR 'microwave radiation'/exp OR 'irreversible electroporation'/exp OR 'stereotactic body radiation therap*':ab,ti,kw OR 'stereotactic body radiotherap*':ab,ti,kw OR 'stereotactic ablative body radiotherap*':ab,ti,kw OR 'stereotactic ablative body radiation therap*':ab,ti,kw OR sbrt:ab,ti,kw OR sabr:ab,ti,kw OR sbr:ab,ti,kw OR 'irreversible electroporation':ab,ti,kw OR ire:ab,ti,kw OR electroporation:ab,ti,kw OR electropermeabilization:ab,ti,kw OR electrocoagulation:ab,ti,kw OR 'microwave near/3 ablati*':ab,ti,kw OR mwa:ab,ti,kw OR rfa:ab,ti,kw OR ((radiofreq*:ab,ti,kw OR 'radio freq*':ab,ti,kw OR thermal*:ab,ti,kw) AND ablat*:ab,ti,kw)).
